# Genome-Wide Identification, Expression Analysis of *HSF* Gene Family in Lanzhou Lily (*Lilium davidii* var. *unicolor*) and Screening of Key Genes *LdHSF10* and *LdHSF40* in Response to High-Temperature Stress

**DOI:** 10.3390/plants15091330

**Published:** 2026-04-27

**Authors:** Qing Yao, Min Mi, Chunmiao Xu, Qingfan Guo, Xinglin Tao, Taohui Fan, Zhaonan Wu, Renmei Dang, Ming Zhao, Yuanxue Yang, Huizhen Ma, Jianye Wei

**Affiliations:** 1 Agricultural Technology Extension Service Center of Linxia Hui Autonomous Prefecture, Linxia 731100, China; 13028770220@163.com (Q.Y.); lxznjfwzx@163.com (Q.G.); lxsmawen@163.com (H.M.); 2 National Key Laboratory of Crop Genetics & Germplasm Innovation and Utilization, Key Laboratory of Biology and Genetic Improvement of Horticultural Crops (East China), Ministry of Agriculture and Rural Affairs of China, Engineering Research Center of Germplasm Enhancement and Utilization of Horticultural Crops, Ministry of Education of China, Nanjing Agricultural University, Nanjing 210095, China; mm@njau.edu.cn; 3 Economic Crops Technology Extension Station of Anding District, Dingxi 743000, China; gslz0721@163.com; 4 Gansu Academy of Agricultural Sciences, Lanzhou 730070, China; taoxinglin77@126.com (X.T.); 18394105722@163.com (R.D.); 5 Linxia Academy of Agricultural Sciences, Linxia 731100, China; 15009302122@163.com (T.F.); 17752268701@163.com (Z.W.); 6 Shandong Academy of Agricultural Sciences, Jinan 250100, China; scrczhm@163.com (M.Z.); yangyuanxue2017@126.com (Y.Y.)

**Keywords:** Lanzhou lily, *HSF*, gene family, *LdHSF10*, *LdHSF40*, high temperature stress response

## Abstract

The heat shock transcription factor (HSF) family is a core regulatory component for plants in response to adversity stress and plays a pivotal role in regulating plant reactions to abiotic stress. Lanzhou lily (*Lilium davidii* var. *unicolor*) is an economically and horticulturally important bulbous crop widely cultivated in Northwest China, and its growth and yield are severely threatened by high-temperature stress during the growing season. Although *HSF* genes have been extensively and thoroughly investigated in other plant species, their functional characterization in lilies remains elusive. In this study, a total of 41 *LdHSF* genes were identified from the genome of *Lilium davidii* var. *unicolor* using bioinformatics approaches. The proteins encoded by these genes exhibited considerable variations in the number of amino acids (aa), as well as distinct isoelectric points (pI) and instability indices. Phylogenetic analysis classified these 41 *LdHSF* genes into three subfamilies (A, B and C). Promoter analysis revealed that the promoters of most *LdHSF* genes were rich in light-responsive *cis*-elements. Meanwhile, the promoters of some genes were highly abundant in hormone-responsive *cis*-elements, whereas those of other genes were enriched in stress-responsive *cis*-elements. Gene expression heatmaps and transcriptomic data demonstrated that the expression patterns of *LdHSF* genes showed significant differences in various tissues and under heat treatment. Based on transcriptomic and RT-qPCR data, we further screened out *LdHSF10* and *LdHSF40* as the major genes responding to heat stress. Functional experiments verified that these two genes encoded nuclear-localized proteins with transcriptional activity. Collectively, these findings lay a solid foundation for elucidating the molecular mechanisms underlying the regulation of heat tolerance by HSF transcription factors (TFs) in lilies in future research.

## 1. Introduction

Lilies are important ornamental plants with high economic value; however, their growth and flowering are highly sensitive to environmental fluctuations, particularly high-temperature stress [1]. Compared with model plants such as *Arabidopsis thaliana*, lilies exhibit a more pronounced susceptibility to heat stress during key developmental stages, including floral initiation and blooming. Heat stress has emerged as a major limiting factor affecting lily production, as it can severely impair vegetative growth, disrupt floral development, and reduce ornamental quality [2]. Recent advances in sequencing technologies have led to the completion of the lily genome, providing valuable resources for dissecting the molecular basis of heat stress responses. However, in contrast to the extensive functional studies conducted in Arabidopsis thaliana and other model species, the regulatory mechanisms underlying heat tolerance in lilies remain largely unexplored [1]. In model plants such as Arabidopsis thaliana, heat stress is known to induce the expression of heat shock proteins (HSPs) and other stress-responsive genes, primarily mediated by heat shock transcription factors (HSFs) [1,3,4,5,6]. Accumulating evidence from studies in Arabidopsis thaliana, rice, and tomato has demonstrated that HSF genes play central roles in establishing thermotolerance; however, whether similar regulatory mechanisms operate in lilies remains unclear [1,7,8,9,10]. Therefore, elucidating the molecular regulatory mechanisms of heat stress responses in lilies, particularly in comparison with well-studied model systems, is of great significance for improving stress tolerance and supporting molecular breeding efforts.

Plants rapidly remodel their transcriptional regulatory networks under stress conditions to maintain cellular homeostasis; this process has been well characterized in *A*. *thaliana* but remains insufficiently studied in lilies [11,12,13,14,15]. Among these regulators, *HSFs* act as key transcription factors controlling stress-responsive gene expression [1,4,16,17,18]. HSF proteins bind specifically to heat shock elements (HSEs) in the promoters of target genes, thereby regulating downstream gene expression; this regulatory mechanism has been extensively validated in *A*. *thaliana*. In addition to heat stress, HSFs in model plants are also involved in multiple stress responses, including drought, salinity, and oxidative stress, as well as hormone signaling pathways; however, such multifunctional roles have not yet been systematically investigated in lilies [19,20,21,22].

Compared with animals and yeast, the *HSF* gene family has undergone significant expansion in plant genomes [23]; for example, *A*. *thaliana* contains more than 20 HSF members, reflecting functional diversification during plant evolution [24,25,26]. Based on structural characteristics, plant *HSFs* are classified into three subclasses (A, B, and C), a classification system that has been well established in *A*. *thaliana* and other species but has not been comprehensively validated in lilies [27]. A typical HSF protein contains a highly conserved DNA-binding domain (DBD) and an oligomerization domain (HR-A/B), and may also harbor functional elements such as the transcriptional activation domain (AHA motif), nuclear localization signal (NLS) and nuclear export signal (NES) [7,8,9,10,11,12,13,14,15,16,17,18]. Among them, members of the *HSFA* subclass mostly act as transcriptional activators to directly induce the expression of downstream stress-responsive genes. Members of the *HSFB* subclass generally lack the AHA activation domain and may function in transcriptional repression and feedback regulation. In contrast, the *HSFC* subclass has a relatively small number of members, and their biological functions remain to be further elucidated [28,29,30,31,32].

In recent years, genome-wide identification and functional analyses of HSF gene families have been extensively conducted in model and crop species such as *A*. *thaliana*, rice, tomato, and cucumber, revealing their critical roles in stress responses and plant development [33]. However, compared with these species, lilies possess large and complex genomes with high repeat content, which has hindered functional genomic studies; consequently, systematic analyses of *HSF* genes in lilies remain limited. The recent release of the lily genome has created new opportunities to investigate the *HSF* gene family at the genome-wide level [34].

Based on this, a systematic identification and comprehensive analysis of the *HSF* gene family was performed at the genome-wide level in lilies in the present study. Through the analysis of physicochemical properties, chromosomal localization, phylogenetic relationships, gene structures and conserved motifs of lily *HSF* genes, combined with the prediction of promoter *cis*-acting elements and the analysis of expression patterns in different tissues or under stress conditions, this study aimed to comprehensively elucidate the evolutionary characteristics of the lily *HSF* gene family and their potential functions in response to abiotic stress. Based on transcriptomic and RT-qPCR data, *LdHSF* genes responsive to heat stress were screened out. Collectively, this study provides an important theoretical basis for further dissecting the heat tolerance regulatory network and mining related functional genes in lilies, and lays a solid foundation for molecular breeding and genetic improvement of stress resistance in lilies.

## 2. Results

### 2.1. Identification of HSF Family Members in Lilium davidii var. unicolor

The *Arabidopsis HSF* genes and protein sequences were used as queries to conduct an initial identification of *HSF* genes in *L*. *davidii* var. *unicolor* via BLAST (version 2.12.0+) and HMMER (version 3.3.2) software. The intersection of the candidate genes identified by the two methods was selected, and a revised model was further constructed for genome-wide prediction of the *HSF* gene family in *L*. *davidii* var. *unicolor*. In total, 41 *HSF* TF genes were identified from the genome of *L*. *davidii* var. *unicolor*. The amino acid (aa) number, isoelectric point (PI), instability index and aliphatic index of LdHSF proteins are shown in Supplementary Table S1. The protein lengths of *LdHSF* genes varied from 99 to 546 aa, with the predicted pI ranging from 4.84 to 10.31, instability indices ranging from 24.73 to 106.26, and aliphatic indices ranging from 54.81 to 103.9. Subcellular localization analysis indicated that most of the predicted LdHSF proteins were localized in the nucleus.

### 2.2. Phylogenetic Analysis of LdHSF Genes

**Figure 1:**
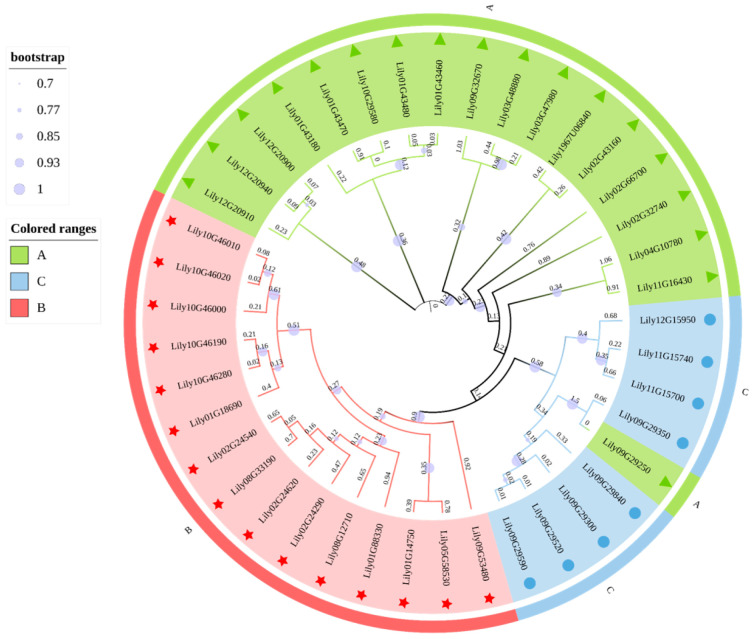
Phylogenetic analysis of LdHSF proteins in *L. davidii* var. *unicolor*. Notes: The green background represents subfamily A, the blue background represents subfamily C, and the red background represents subfamily B. The star, triangle, and circle symbols mark members of different subfamilies, respectively.

To investigate the evolutionary relationships among *HSF* genes in *L*. *davidii* var. *unicolor*, multiple sequence alignment of the identified LdHSF protein sequences was performed using MUSCLE (version 3.8.31) software. A phylogenetic tree was subsequently constructed with the Maximum Likelihood (ML) method and JTT model via FastTree (version 2.1.11) software (Figure 1). All the 41 identified LdHSF proteins were phylogenetically clustered into three distinct subfamilies, namely A, B, and C. Among them, the *HSFB* subfamily contained the largest number of members with 20 proteins, while the *HSFC* subfamily had the fewest members with only eight proteins. The *HSFA* and *HSFC* subfamilies shared a more recent ancestral node, indicating a relatively close genetic relationship between them. In contrast, the *HSFB* subfamily formed an independent evolutionary clade, which suggested that it had undergone an earlier functional differentiation or independent gene expansion event during the evolution of the *HSF* gene family.

### 2.3. Motif and Gene Structure Analyses of LdHSF Genes

**Figure 2:**
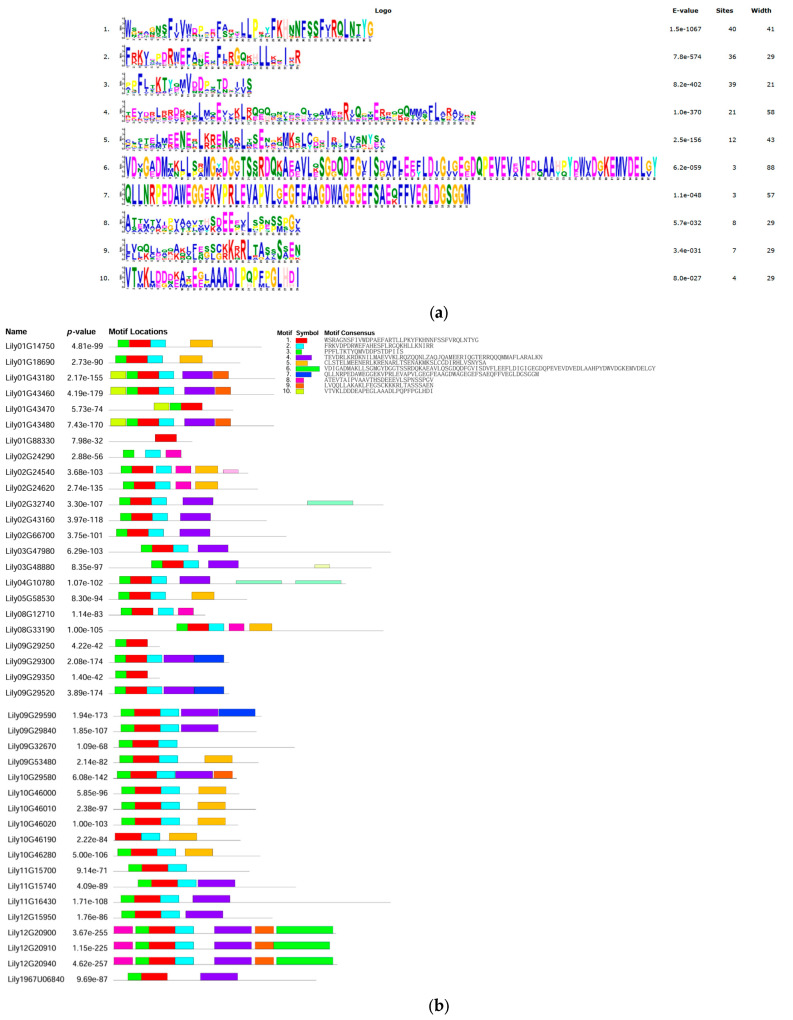

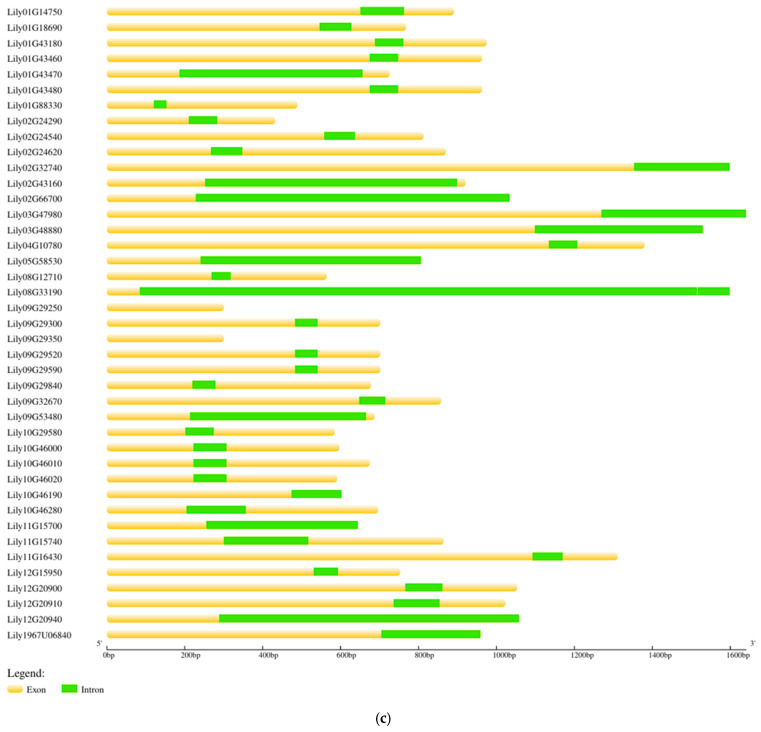
Conserved motif and gene structure analyses of the *LdHSF* gene family in *L. davidii* var. *unicolor*. Notes: (**a**) Sequence logos of the 10 conserved motifs identified in LdHSF proteins. The height of each amino acid residue at a given position reflects its conservation level, with larger letters indicating higher conservation. The E-value, number of target sites, and width of each motif are listed on the right. (**b**) Schematic distribution of the 10 conserved motifs in individual LdHSF proteins. Each colored box represents a specific motif (corresponding to the motif logos in panel a), and the order of boxes corresponds to the position of the motif in the protein sequence. (**c**) Exon–intron structure of *LdHSF* genes. Yellow boxes represent exons, green boxes represent introns, and the horizontal lines represent the full-length gene sequences. The scale bar at the bottom indicates the physical length of the genes (in base pairs, bp).

The types and compositions of conserved motifs are usually closely associated with protein functions and evolutionary relationships. To characterize these features in LdHSF proteins, a total of 10 conserved motifs were identified and analyzed in the 41 LdHSF proteins (Figure 2a,b). Among the ten detected motifs, motif 1, motif 2 and motif 3 were highly conserved across the *LdHSF* family; motif 1 was present in all identified LdHSF proteins. The sequential combination of motif 3, motif 1 and motif 2 was observed in the vast majority of *LdHSF* genes, implying that this combination constitutes the core structural domain of LdHSF proteins. Notably, motif 8 was uniquely present in *Lily02G24540*. Overall, LdHSF proteins within the same subfamily exhibited similar motif compositions, which may indicate the conservation of their structural and functional characteristics. Gene structure analysis of *LdHSF* genes was also performed (Figure 2c), and the results showed that the number of exons in *LdHSF* genes ranged from 1 to 2, with intron numbers varying from 0 to 2. Most *LdHSF* genes with close genetic relationships shared similar gene structural patterns.

### 2.4. Prediction of Cis-Acting Elements in the Promoters of LdHSF Genes

**Figure 3:**
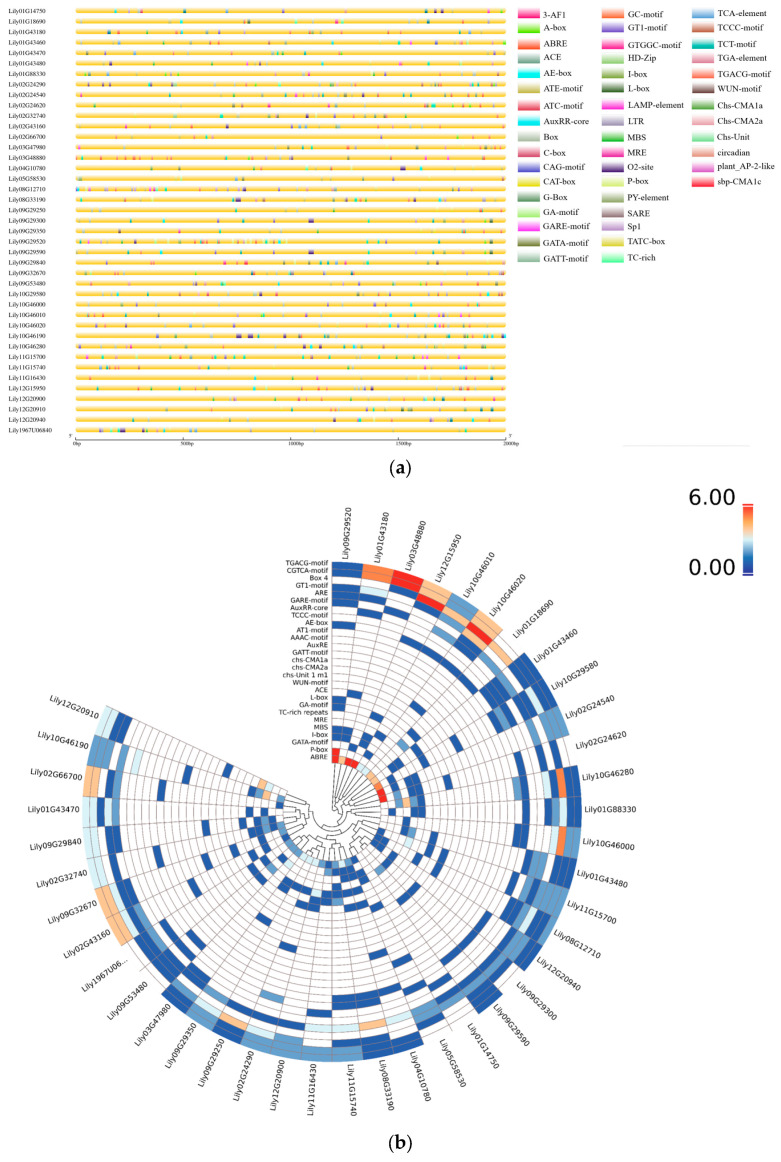
Prediction and analysis of cis-acting elements in the promoter regions of *LdHSF* genes in *L. davidii* var. *unicolor*. (**a**) Schematic distribution of predicted *cis*-acting elements in the 2000 bp upstream promoter regions of *LdHSF* genes. Each colored vertical line represents a specific *cis*-acting element, with the legend on the right indicating the correspondence between colors and element types. The horizontal axis shows the physical position of the promoter region from the 5′ to 3′ end, with the scale bar indicating length in base pairs (bp). (**b**) Circular heatmap visualizing the occurrence frequency of different *cis*-acting elements in the promoters of each *LdHSF* gene. The color gradient (from blue to red) represents the number of element occurrences, with red indicating higher frequency and blue indicating lower frequency. The outer ring labels correspond to individual *LdHSF* genes, and the inner ring labels represent the types of *cis*-acting elements.

To explore the potential functions and regulatory mechanisms of *LdHSF* genes, the 2000 base pair (bp) sequences upstream of the coding regions of the 41 identified *LdHSF* genes were extracted as promoter regions, and the *cis*-acting elements within these regions were further predicted to elucidate the transcriptional regulatory mechanisms of *LdHSF* genes in *L*. *davidii* var. *unicolor* (Figure 3a). These identified *cis*-acting elements were classified into five categories: hormone-responsive elements, environmental stress-responsive elements, light-responsive elements, development and metabolism regulatory elements, and other functional elements. For further investigation, the occurrence frequencies of the first four categories of *cis*-acting elements in *LdHSF* gene promoters were statistically analyzed and visualized as a heatmap (Figure 3b). The composition of promoter *cis*-acting elements among *LdHSF* family members exhibited significant diversity and specificity. Most *LdHSF* gene promoters were enriched with light-responsive elements (e.g., GT1-motif), suggesting that light signaling may represent one of the core regulatory pathways for the *LdHSF* gene family. Meanwhile, the promoters of some *LdHSF* genes (e.g., *Lily01G43460*) were highly abundant in hormone-responsive elements (e.g., ABRE), whereas the promoters of other genes (e.g., *Lily09G29350*) were enriched with stress-responsive elements (e.g., MBS). These results indicated that functional differentiation has occurred in the expression regulatory patterns among different members of the *LdHSF* gene family. In terms of element abundance, light-responsive elements showed the highest average occurrence frequency across the *LdHSF* family. By contrast, hormone-responsive and stress-responsive elements displayed the characteristic of member-specific high abundance, which reflected the functional specialization of *LdHSF* family members in hormone response and environmental adaptation. The distribution patterns of these *cis*-acting elements provide a critical basis for subsequent dissection of the expression regulatory network and functional differentiation mechanisms of the *LdHSF* gene family, and also imply that *LdHSF* members may perform synergistic yet divergent roles in plant growth and stress resistance processes.

### 2.5. Chromosomal Localization of LdHSF Genes

**Figure 4:**
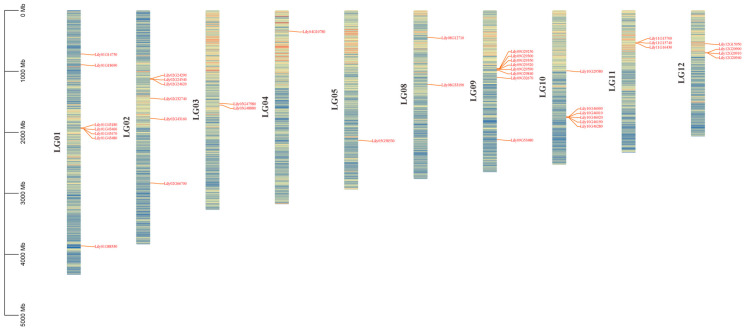
Chromosomal localization of the *LdHSF* gene family in *L. davidii* var. *unicolor*. The 12 pseudochromosomes (LG01–LG12) and one unanchored contig (Contig19967) of the lily genome are represented by vertical bars, with the scale on the left indicating the physical length of the chromosomes in megabase (Mb). The red labels and connecting lines mark the exact physical positions of each *LdHSF* gene on the corresponding chromosomes or contig, showing the distribution pattern of the *LdHSF* gene family across the lily genome.

To determine the physical locations of *LdHSF* genes in the *L. davidii* var. *unicolor* genome, chromosomal localization analysis was performed for the identified *HSF* genes. A total of 41 *LdHSF* genes were mapped to 12 chromosomes and one unanchored contig (contig1967) (Figure 4). Chromosomes 1, 2, 9 and 10 contained a relatively high number of *LdHSF* genes, with approximately six to eight members distributed on each of these chromosomes. In contrast, only a single *LdHSF* gene was localized on chromosome 4 and chromosome 5, respectively. Overall, *LdHSF* genes exhibited an extremely uneven distribution across the lily chromosomes, and no significant correlation was observed between the number of *LdHSF* family members and chromosome length.

### 2.6. Tissue Expression Specificity and Expression Pattern Analysis of LdHSF Genes Under Heat Treatment

**Figure 5:**
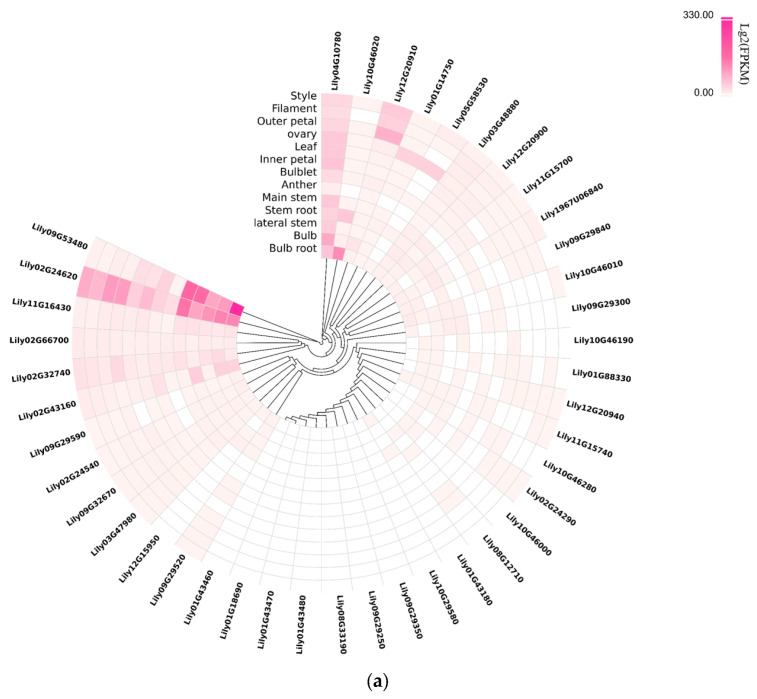

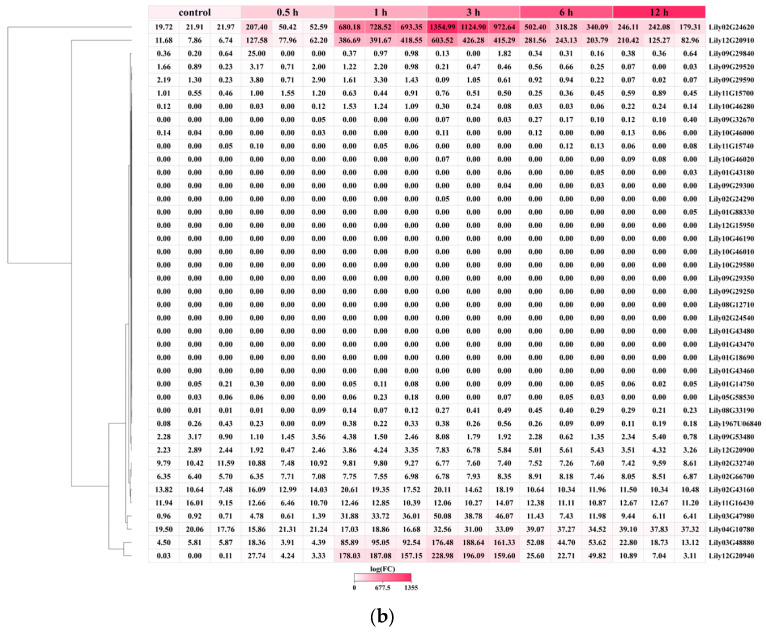
Expression profiles of *LdHSF* genes in *L. davidii* var. *unicolor*. (**a**) Circular heatmap showing the tissue-specific expression patterns of *LdHSF* genes. The outer ring labels represent individual *LdHSF* genes, and the inner ring labels indicate the sampled tissues (including bulb, bulb root, stem, leaf, flower organs, etc.). The color gradient (from white to deep pink/red) represents the normalized log_2_^(FPKM)^ values, with darker red indicating higher expression levels. (**b**) Heatmap illustrating the dynamic expression changes in *LdHSF* genes in leaves under heat stress. The horizontal axis shows different heat treatment time points (control, 0.5 h, 1 h, 3 h, 6 h, 12 h), and the vertical axis lists individual *LdHSF* genes. The color scale (ranging from white to red) represents the absolute expression levels of *LdHSF* genes, where red indicates higher expression and white indicates lower or no expression.

The expression profiles of *LdHSF* genes in different tissues of *L*. *davidii* var. *unicolor* were analyzed based on transcriptomic data (Figure 5a). Some *LdHSF* genes exhibited constitutively high expression across all tested tissues: *Lily02G24620* was highly expressed in all tissues, with relatively higher expression levels in the main stem and bulb. Compared with other tissues, *Lily09G53480* showed extremely high expression in bulb roots and the main stem, representing a dominantly expressed gene in bulb roots. *Lily04G10780* maintained a high expression level in tissues including bulbs and ovaries. Meanwhile, several other genes displayed tissue-specific expression preferences in single or a few tissues: *Lily01G14750* was highly expressed only in ovaries with extremely low expression levels in other tissues, while *Lily12G20910* showed significantly higher expression in outer petals and filaments than in other tissues. In addition, more than half of the *LdHSF* genes exhibited zero or near-zero expression levels in all detected tissues, which suggested that these genes might be activated for expression under specific stress conditions or possess functional redundancy.

To investigate the responses of *LdHSF* genes to heat stress, their expression patterns under heat treatment were further analyzed using the transcriptome dataset SRP304395 (Figure 5b). Most *LdHSF* genes showed near-zero expression levels at all detected time points, with no obvious responses to heat treatment. *Lily02G24620* and *Lily12G20910* responded rapidly to heat treatment at 0.5 h–1 h and reached their expression peaks after 3 h of treatment. The expression levels of *Lily03G48880*, *Lily12G20940* and *Lily03G47980* increased significantly at 1 h post heat treatment. *Lily04G10780* exhibited a relatively slow response to heat stress, with its expression level gradually increasing as the treatment duration extended. Interestingly, among the *LdHSF* genes responsive to heat stress, the expression levels of most genes gradually decreased after 6–12 h of treatment, whereas the expression level of *Lily04G10780* continued to show an increasing trend.

### 2.7. Validation of LdHSF Gene Responses to Heat Stress by RT-qPCR

**Figure 6:**
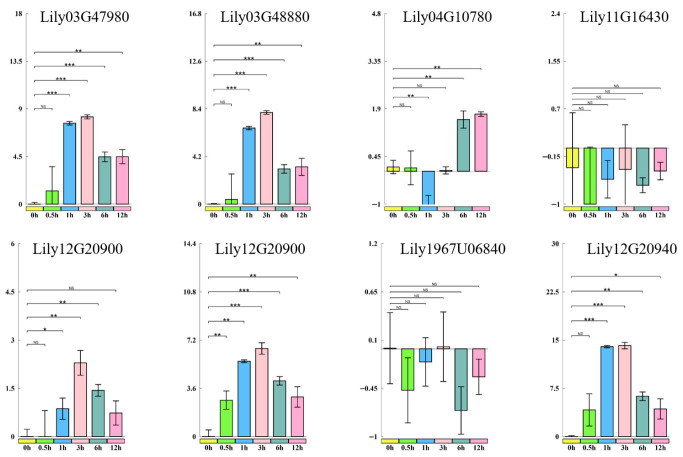
Relative expression levels of 12 LdHSF genes at 0 h (control), 0.5 h, 1 h, 3 h, 6 h, and 12 h after heat treatment were detected by RT-qPCR. The lily 18S rRNA gene was used as the internal reference. The expression values were calculated using the 2^−ΔΔCT^ method and log_2_-transformed for visualization. Different colors represent different time points: 0 h (yellow), 0.5 h (green), 1 h (blue), 3 h (light red), 6 h (cyan), and 12 h (pink). Error bars represent the standard deviation (SD) of three biological replicates. Significant differences compared to the 0 h control are indicated with asterisks (* *p* < 0.05, ** *p* < 0.01, *** *p* < 0.001, NS: not significant (*p* > 0.05)).

Based on the tissue expression specificity analysis and expression pattern analysis of *LdHSF* genes under heat treatment, the *LdHSF* genes with heat stress-responsive expression were selected for RT-qPCR analysis (Figure 6). The results showed that the expression levels of *Lily02G24620*, *Lily03G47980*, *Lily12G20940* and *Lily12G20910* increased rapidly at 0.5 h post heat treatment, while the expression levels of *Lily02G48880* and *Lily12G20900* were sharply upregulated at 1 h post heat treatment. The expression trends of the 12 selected *LdHSF* genes in the RT-qPCR assays were consistent with those in the transcriptomic data. *Lily02G24620* and *Lily12G20940* were selected as the candidate genes for further research. According to their chromosomal localization positions, *Lily02G24620* and *Lily12G20940* were designated as *LdHSF10* and *LdHSF40*, respectively.

### 2.8. LdHSF10 and LdHSF40 Are Heat-Responsive Nuclear-Localized TFs

**Figure 7:**
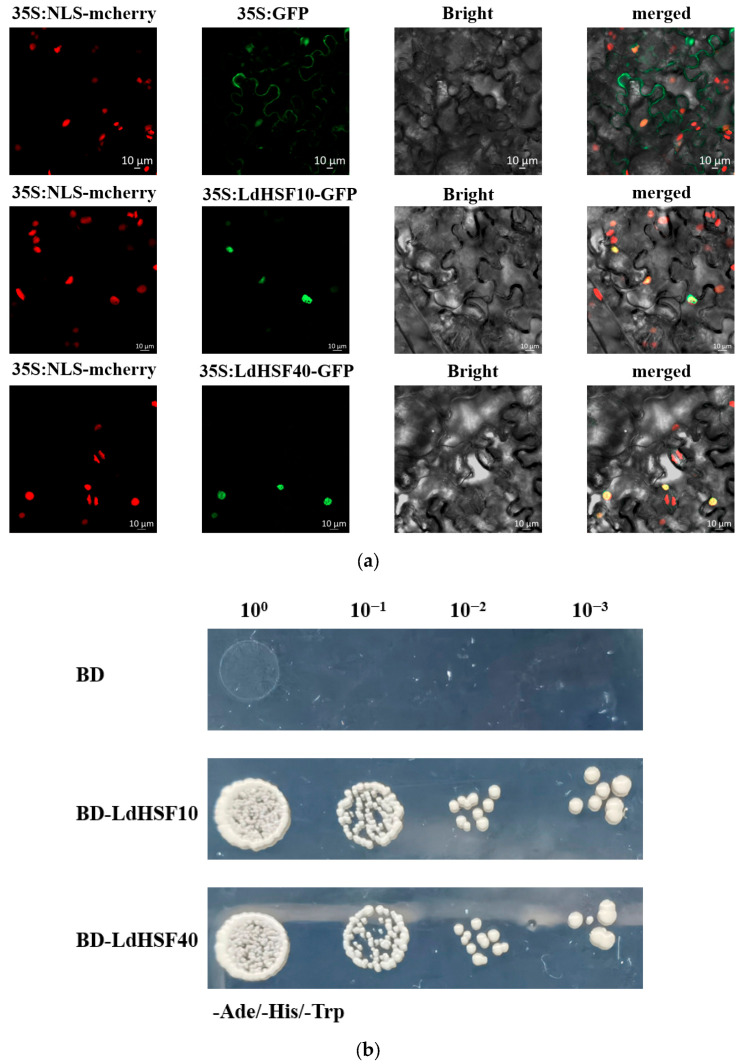
(**a**) The subcellular localization of the *LdHSF* genes and the empty vector tagged with GFP and transiently expressed in *N. benthamiana*. (**b**) Transcriptional Activity Assay of *LdHSF10* and *LdHSF40* in Yeast (SD/-Ade/-His/-Trp Medium). Yeast cells were spotted on -Ade/-His/-Trp selective medium in 10-fold serial dilutions (10^0^, 10^−1^, 10^−2^, 10^−3^).

Transcriptomic data and RT-qPCR analyses demonstrated that the expression levels of *LdHSF10* and *LdHSF40* were rapidly upregulated in lily leaf tissues after heat treatment (Figure 5b and Figure 6). To further investigate the molecular functions of *LdHSF10* and *LdHSF40*, their subcellular localization was first determined via the tobacco transient expression system. The results showed that *LdHSF10* and *LdHSF40* were predominantly localized in the nucleus (Figure 7a,b). To verify whether *LdHSF10* and *LdHSF40* possessed transcriptional activation activity, a yeast transactivation assay was performed, with the empty vector pGBKT7 used as the negative control. The results indicated that yeast cells harboring BD-LdHSF10 and BD-LdHSF40 fusion vectors were able to grow normally on the SD/-Trp/-His selective medium, whereas yeast cells transformed with the empty pGBKT7 vector failed to grow on the same medium (Figure 7b). Collectively, these results confirmed that *LdHSF10* and *LdHSF40* function as heat-responsive nuclear-localized TFs in lilies.

## 3. Discussion

As a core regulatory component for plants to respond to adverse stresses, the structural characteristics and functional differentiation of the HSF family have always been research hotspots in the field of plant molecular biology [3,35,36,37]. Members of the *HSF* family generally possess highly conserved core structural domains, such as the N-terminal DNA-binding domain (DBD), oligomerization domain (OD/HR-A/B), and NLS. The specific variations in these structures are the molecular basis for their functional differentiation [38]. According to the sequence differences in the oligomerization domain (OD), the plant *HSF* family can be divided into three major subfamilies: A, B, and C. Among them, members of the *HSFA* subfamily possess transcriptional activation ability due to the C-terminal AHA activation motif they contain, while most members of the HSFB subfamily exert transcriptional regulatory functions through the conserved -LFGV- repression motif. However, the function of the *HSFC* subfamily has not yet been fully elucidated [26,39]. There is a significant difference in the size of the *HSF* family among different species. For example, *A*. *thaliana* contains 21 *HSF* members [40], whereas wheat has as many as 56 members [41]. This quantitative divergence is likely closely associated with the adaptive evolution of species in response to environmental stresses [42]. Based on the lily genome from Nanjing Agricultural University, we identified 41 *LdHSF* genes, which were divided into A, B, and C subfamilies. These *LdHSF* genes were not evenly distributed in the genome, and chromosomes 1, 2, 9 and 10 contained more *LdHSF* genes. The uneven distribution of *LdHSF* genes on chromosomes indicated that there are diverse structural characteristics within the *HSF* gene family [43,44].

The LdHSF proteins exhibited significant differences in physicochemical properties, with their aa lengths ranging from 99 to 546 residues, predicted pI spanning 4.84 to 10.31, instability indices varying from 24.73 to 106.26, and aliphatic indices ranging from 54.81 to 103.9. Subcellular localization prediction analysis indicated that all the identified LdHSF proteins were localized in the nucleus, which was consistent with the subcellular localization predictions of HSF proteins in other plant species [45,46].

The *HSF* family plays multiple roles in plant responses to heat stress. Most members of the *HSFA* subfamily act as positive regulators which activate the expression of heat shock protein (*HSP*) genes to maintain protein homeostasis by binding to the HSEs in the promoter regions of downstream *HSP* genes [6,47,48]. As a typical example, the heterologous overexpression of wheat *TaHSF3* confers enhanced thermotolerance in transgenic *A*. *thaliana* [49]. In contrast, members of the Arabidopsis *HSFA1* subfamily serve as master regulators of heat stress responses, and their quadruple mutant completely loses acquired thermotolerance [50]. Notably, some members of the *HSFB* subfamily exhibit functional diversity. Overexpression of the garlic *AsHSFB1* gene reduces the antioxidant enzyme activity and chlorophyll content in transgenic *A*. *thaliana* while increasing the malondialdehyde (MDA) level, indicating that it exerts a negative regulatory role in plant heat stress responses [18]. By contrast, wheat *TaHSFB4-2B* enhances plant tolerance to abiotic stresses via modulating the expression of stress-responsive defense genes [51]. This functional divergence may be attributed to the evolution of species-specific regulatory networks.

Prediction analysis of *cis*-acting regulatory elements revealed that members of the *LdHSF* gene family contained a variety of light-responsive, hormone-responsive and abiotic stress-responsive elements. These results indicated that *LdHSF* genes might be involved in multiple processes related to plant growth and development as well as stress adaptation. The regulatory mechanisms of the *HSF* family exhibit multi-level complexity. At the transcriptional regulation level, *HSF* members can regulate the expression of target genes by forming homo- or hetero-oligomers. For example, the hetero-oligomer formed by tomato *SlHSFA1a* and *SlHSFA2* can significantly enhance the transcriptional activation efficiency of downstream target genes [52]. Meanwhile, the cross-regulation between *HSFs* and other transcription factors further enriches the regulatory network. For instance, the expression of *Arabidopsis HSFA3* is directly regulated by the *DREB2A* TF, forming a cross-pathway in response to drought–heat stress [53]. At the post-translational regulatory level, the *HSP90/HSP70* chaperone complex maintains the inactive state of HSF through physical interaction with *HSF*; upon heat stress, dissociation of this complex triggers the activation and nuclear localization of *HSF* [54]. In contrast, SUMOylation can suppress the transcriptional activity of *HSFA2* [55]. Furthermore, recent studies have revealed that *HSF-1* regulates the lifespan of *Caenorhabditis elegans* via ubiquilin-1-dependent mitochondrial network remodeling, which uncovers a novel function of the *HSF* family in the regulation of energy metabolism and aging [56].

Expression analysis of the *LdHSF* genes showed that among the 41 identified *LdHSF* genes, 29 were not detected or exhibited extremely low expression levels in different tissues of lily. Previous studies have demonstrated that the lily genome, which is composed of repetitive sequences, accounts for up to 88.31% of the total genome, and the proportion of long terminal repeat (LTR) retrotransposons reaches 64.6%, a value that is higher than that of most plant species [1]. We thus reasonably speculate that these insertions have disrupted the structural integrity of certain *HSF* genes, thereby inducing transcriptional silencing and potential loss of their functions. The expression patterns and functional divergence of the *HSF* family across different species reflect their adaptive evolutionary characteristics. Analysis of the tissue-specific expression of 22 *HSF* genes in garlic revealed that members of the subfamily B, such as *Asa2G05473.1*, were highly expressed in bulbs, implying that they may be involved in the coordinated regulation of bulb development and stress tolerance [18]. Among the 18 *AoHSF* genes in asparagus, members including *AoHsf05* and *AoHsf13* exhibited significant responses to heat, cold and salt stress in root tissues, which provides candidate genes for the breeding of multi-stress tolerance [57]. The expression of the wheat *TaHSFA6e* gene was significantly higher in the heat-tolerant cultivar Halna than in heat-sensitive cultivars, and its expression level was positively correlated with the transcriptional activation of target genes such as *HSP17* and *HSP70* as well as the activity of antioxidant enzymes [58]. This indicates that the differential expression of *HSF* genes may act as a key factor underlying the divergence in heat tolerance among wheat cultivars.

In the present study, we identified 41 *LdHSF* genes that respond to heat stress using transcriptome data and RT-qPCR and screened out *LdHSF10* and *LdHSF40*. Combining transcriptome data with RT-qPCR, we initially confirmed through yeast experiments and tobacco transient transformation assays that *LdHSF10* and *LdHSF40* are heat-responsive nuclear-localized TFs. In subsequent studies, we will focus on the regulatory networks involving *LdHSF10*, *LdHSF40,* and their downstream targets, and clarify the specific regulatory mechanisms through further genetic and molecular studies.

## 4. Materials and Methods

### 4.1. Identification of LdHSF Genes

The genome sequence of *L*. *davidii* var. *unicolor* was downloaded from the Liliales Genome Database developed by the Lily Lab of Nanjing Agricultural University, China [59] (https://lgd.njau.edu.cn/lily/home (accessed on 14 December 2025)). To identify the *HSF* genes in *L*. *davidii* var. *unicolor*, we first downloaded the *HSF* gene and protein sequences of *A*. *thaliana* from the Arabidopsis Information Resource (TAIR) database [60] (https://www.arabidopsis.org/ (accessed on 16 December 2025)), and then obtained the HMM model (PF00447) of the HSF family from the Pfam database [61] (https://www.ebi.ac.uk/interpro/entry/pfam/ (accessed on 15 December 2025)). A preliminary identification of *HSF* genes in *L*. *davidii* var. *unicolor* was performed using BLAST and HMMER software with the *HSF* gene and protein sequences of *Arabidopsis thaliana* as references. After taking the intersection of the results from the two identification methods, a new model was constructed for the genome-wide prediction of the *HSF* gene family in *L*. *davidii* var. *unicolor*. The ExPASy ProtParam tool [62] (https://web.expasy.org/protparam/ (accessed on 17 December 2025)) was employed to predict the aa length, molecular weight (MW), theoretical pI, instability index, and aliphatic index of the identified HSF proteins. Additionally, the Plant-mPLoc server [63] (http://www.csbio.sjtu.edu.cn/bioinf/plant-multi/ (accessed on 19 December 2025)) was used to predict the subcellular localization of LdHSF proteins.

### 4.2. Construction of Phylogenetic Tree of LdHSF Genes

Multiple sequence alignment of the identified HSF proteins from *L*. *davidii* var. *unicolor* was first performed using MUSCLE. Subsequently, a phylogenetic tree was constructed with FastTree software [64] based on the ML method and the JTT substitution model. The generated phylogenetic tree was further optimized and visualized using the Interactive Tree of Life (iTOL) online tool [65] (https://itol.embl.de/ (accessed on 22 December 2025)). According to the BLAST results and the subfamily classification of reference sequences, the identified members of the *HSF* gene family were classified into corresponding subgroups.

### 4.3. Prediction of Motifs and Analysis of Gene Structures of LdHSF Genes

Motif prediction of the identified *HSF* genes was conducted using the MEME suite (Multiple Em for Motif Elicitation), and custom R scripts were applied to generate motif SeqLoga plots. Information regarding the members of the *HSF* gene family was extracted from the genome annotation file of *L*. *davidii* var. *unicolor*, and the Gene Structure Display Server (GSDS) online platform [66] (https://gsds.gao-lab.org/index.php (accessed on 24 December 2025)) was utilized to visualize the gene structures.

### 4.4. Prediction of Cis-Acting Elements in the Promoters of LdHSF Genes

According to the downloaded genome annotation files, the 2000 bp upstream sequences of the identified *HSF* genes were extracted as promoter regions for the prediction of cis-acting elements. The online tool Plant CARE was used to predict *cis*-acting elements in the extracted promoter sequences. After filtering the predicted results, TBtools (version 1.127) software [67] was employed to visualize the distribution of *cis*-acting elements.

### 4.5. Chromosomal Localization of LdHSF Genes

Based on the genome annotation files, TBtools (version 1.127) software [67] was used to analyze and visualize the chromosomal and contig positions of the identified *HSF* genes.

### 4.6. Tissue-Specific Expression Analysis and Heat Stress-Induced Expression Pattern Analysis of LdHSF Genes Using Transcriptome Data

Tissue-specific expression analysis of *L*. *davidii* var. *unicolor HSF* genes was performed using tissue expression-level data retrieved from the Liliales Genome Database [63] developed by the Lily Lab of Nanjing Agricultural University, China (https://lgd.njau.edu.cn/lily/home (accessed on 26 December 2025)). For the analysis of *HSF* gene expression patterns under heat treatment, publicly available transcriptome data (SRP304395) were downloaded from the National Center for Biotechnology Information (NCBI) database. Gene expression levels across various tissues and under different temperature treatments were normalized as log_2_(TPM + 1), and custom R scripts were used to generate heatmaps for visualization.

### 4.7. Plant Materials and Growth Conditions

*L*. *davidii* var. *unicolor* were used as experimental materials in this study. Lily plantlets were propagated on Murashige and Skoog (MS) medium and subcultured every two weeks under controlled conditions (22 °C, 16 h light/8 h dark photoperiod) in a standard culture room. *N*. *benthamiana* (tobacco) seeds were surface-sterilized and germinated on MS medium. Ten days after germination, seedlings were transplanted into individual plastic pots and grown in an incubator

### 4.8. RT-qPCR Analysis of LdHSF Expression Patterns Under Heat Stress

Two-week-old, healthy *L*. *davidii* var. *unicolor* tissue-cultured seedlings of uniform size were subjected to heat stress at 42 °C for various durations (0, 0.5, 1, 3, 6, and 12 h) in a temperature-controlled incubator (DRP-9082, Shanghai, China). After each treatment, 2–3 leaves were harvested for total RNA extraction, and the expression levels of genes were quantified using RT-qPCR. The RT-qPCR cycling conditions were as follows: 95 °C for 30 s; 40 cycles of 95 °C for 5 s, 60 °C for 30 s, and 95 °C for 15 s; and a final melting curve analysis consisting of 65 °C for 60 s and 95 °C for 15 s. The lily 18S rRNA gene was used as an internal reference for normalization [1], and relative gene expression levels were calculated using the 2^−ΔΔCT^ method. For statistical analysis and result visualization, the quantitative 2^−ΔΔCT^ values were subjected to log_2_ transformation.

### 4.9. Subcellular Localization Analysis

The coding sequence (CDS) regions of *LdHSF10* and *LdHSF40* genes, with stop codons removed, were inserted into the pCAMBIA1302 vector to construct recombinant plasmids pCAMBIA1302-*LdHSF10* and pCAMBIA1302-*LdHSF40*. The empty pCAMBIA1302 vector, pCAMBIA1302-*LdHSF10*, and pCAMBIA1302-*LdHSF40* were individually transformed into *Agrobacterium tumefaciens* GV3101 competent cells. The transformed agrobacterial cells were then cultured in liquid LB medium at 28 °C with shaking at 200 rpm for 16–24 h for subsequent use.

Bacterial cells were harvested by centrifugation at 6000 rpm for 10 min, and the pellets were resuspended in infiltration buffer (10 mM MES (Coolaber, Beijing, China), 10 mM MgCl_2_ (Coolaber, Beijing, China), and 150 μM acetosyringone (As) (Coolaber, Beijing, China). The optical density at 600 nm (OD_600_) of the bacterial suspension was adjusted to 0.8. After incubation at room temperature in the dark for 3–4 h, the suspension was infiltrated into *N*. benthamiana leaves expressing mCherry nuclear localization signal (mCherry-NLS). The infiltrated tobacco plants were cultured in darkness for 12 h, followed by a 36–60 h incubation under light conditions.

Using the empty pCAMBIA1302 vector as the control, the fluorescence signals in tobacco leaf cells were detected and imaged using a high-resolution confocal laser scanning microscope (LSM900, Carl Zeiss, Oberkochen, Germany).

### 4.10. Transcriptional Activation Activity Assay of TFs

The CDS of *LdHSF10* and *LdHSF40* were amplified with the stop codons removed, and inserted into the pGBKT7 vector (Clontech/Takara, Kusatsu, Japan). The recombinant plasmids were then transformed into the yeast strain Y2HGold (Coolaber, Beijing, China). The transformed cells were spread on SD/-Trp (Coolaber, Beijing, China) deficient medium and incubated upside down at 28 °C for 3–4 days.

Single yeast colonies were picked and diluted with sterile water to an OD_600_ of 0.2, 0.02, 0.02, and 0.0002, respectively. The dilutions were spotted onto SD/-Ade/-His/-Trp (Coolaber, Beijing, China) selective medium. After incubation at 28 °C for 2–3 days, the growth status of yeast cells was observed and recorded.

## Data Availability

The data presented in this study can be obtained from the corresponding author upon reasonable request.

## Supplementary Materials

The following supporting information can be downloaded at: https://www.mdpi.com/article/10.3390/plants15091330/s1, Table S1: Physicochemical properties and subcellular localization of the identified LdHSF proteins; File S1: lily.HSF.cds.fasta, the coding sequence (CDS) file of *LdHSF* genes; File S2: lily.HSF.pep.fasta, the protein sequence file of *LdHSF* genes.
